# Salivary carcinoembryonic antigen in desquamative gingivitis: A comparative study in oral lichenoid reactions before and after topical corticosteroid therapy

**DOI:** 10.34172/japid.2025.004

**Published:** 2025-01-20

**Authors:** Ayla Bahramian, Farzaneh Pakdel, Solmaz Pourzare Mehrbani, Ehsan Golchin, Ensiyeh Maljaei, Maryam Hosseinpour Sarmadi, Tara Deljavanghodrati, Katayoun Katebi

**Affiliations:** ^1^Department of Oral and Maxillofacial Medicine, Faculty of Dentistry, Tabriz University of Medical Sciences, Tabriz, Iran; ^2^Toxoplasmosis Research Center, Mazandaran University of Medical Science, Sari, Iran; ^3^Private Practice, Tabriz, Iran; ^4^Dentistry Faculty of Yeditepe University, Istanbul, Turkey

**Keywords:** Carcinoembryonic antigen, Oral lichenoid reactions, Saliva

## Abstract

**Background.:**

Desquamative gingivitis is an immunological chronic disease that is considered precancerous and has the potential to develop into squamous cell carcinoma. Carcinoembryonic antigen (CEA), a common tumor marker, increases in many cancers. The present study compared salivary CEA levels in desquamative gingivitis before and after topical corticosteroid therapy.

**Methods.:**

This case‒control study was conducted in the Department of Oral and Maxillofacial Medicine, Tabriz Faculty of Dentistry. Twenty patients with desquamative gingivitis in oral lichen planus (OLP) background were selected as the case group, with 20 healthy individuals as the control group. Desquamative gingivitis lesions were confirmed with biopsies. Salivary samples were obtained from both groups. Second, salivary samples were collected from the case group after a course of topical corticosteroid therapy. Salivary CEA levels were measured by a monobind kit using the ELISA method. Independent and paired t-tests were used to analyze the data in SPSS 17. *P*<0.05 was considered statistically significant.

**Results.:**

Before treatment, CEA levels were significantly higher in the case group (174.06±95.55) than in the control group (55.66±41.26 ng/mL) (*P*<0.001). Salivary CEA levels in the case group decreased significantly after the treatment (96.77±66.25 ng/mL) compared to before treatment (174.06±95.55 ng/mL) (*P*<0.001).

**Conclusion.:**

This study demonstrated that CEA levels significantly decreased in patients with desquamative gingivitis associated with oral lichenoid reaction after receiving topical corticosteroid therapy.

## Introduction

 Oral lichenoid reactions (OLRs) are a group of oral lesions with characteristic clinical and histological manifestations. These reactions include oral lichen planus (OLP), oral lichenoid contact reaction, oral lichenoid drug reaction, and lichenoid reaction induced by graft-versus-host disease (GvHD).^1^ OLP is a chronic inflammatory condition involving the oral mucosa and gingiva and is believed to be an autoimmune T-cell-mediated disease. One of the most prevalent presentations of lichenoid reactions is desquamative gingivitis. Its underlying etiology has not yet been fully understood.^2^ Several studies have reported a relatively high prevalence for this disease, with almost 0.5%‒2.5% of the general population affected. Moreover, it has been shown that the disease is more common in the middle-aged population and females.^3,4^

 Desquamative gingivitis is a nonspecific clinical manifestation that can occur in various pathological conditions. It is most frequently observed alongside mucocutaneous disorders, such as OLRs, mucous membrane pemphigoid, and pemphigus vulgaris. The pain and discomfort caused by desquamative gingivitis can discourage patients from effectively brushing their teeth, which may increase the risk of long-term periodontal tissue damage due to plaque buildup in specific areas.^5^ There is a risk of malignant transformation associated with desquamative gingivitis in the context of OLRs, which occurs in 0.4%‒5.3% of cases.^6,7^

 The gold standard for oral cancer diagnosis is tissue biopsy and subsequent histopathological examination. However, this method is invasive, painful, time-consuming, and costly.^8^ Oral cancer research has recently introduced more effective tools for early diagnosis of this problem, including brush biopsy, toluidine blue staining, and saliva investigations, which may result in early detection of oral dysplasia. However, each technique has specific Early detection of precancerous and malignant lesions of the oral mucosa, which can improve the prognosis and survival of the patients.^9^

 Due to a lack of distinguishing clinical manifestations, oral cancer is often diagnosed in late stages.^10,11^ Various salivary and serum biomarkers have been noted to detect dysplastic precancerous lesions and malignancies early. Cheng et al^12^ investigated the tumor marker endothelin-1 in the saliva of patients with lichen planus, introducing this biomarker as a lichen planus activity assessment tool.

 Another biomarker, a glycoprotein involved in cellular adhesion known as the carcinoembryonic antigen (CEA), is one of the most common tumor markers, and its levels have been evaluated in many malignancies. The levels of this biomarker increase in several malignancies. However, despite the expression of CEA in malignant tissues, its serum levels are normal in some cancers.^13^ This biomarker can indicate the progression or regression of malignant diseases. Therefore, it can be used for early detection of disease recurrence and cancer treatment monitoring.^14^

 Nowadays, the use of saliva as a diagnostic tool has attracted the attention of many researchers since saliva collection is a simple and non-invasive method.^15^ According to He et al,^16^ CEA levels significantly increase in oral Squamous cell carcinoma (SCC). Zheng et al investigated the serum and salivary CEA levels in precancerous and cancerous lesions, including OLP, leukoplakia, and squamous cell carcinoma, showing an increased level of CEA in serum and saliva in patients with malignancy.^17^ Also, Li et al,^18^ investigated the CEA levels in patients with oral SCC. They showed that CEA levels significantly increased in saliva and cells that had been locally peeled from the tumor, suggesting the use of CEA as a reliable marker for early detection of malignant oral cancers.

 CEA levels in the serum of patients with SCC have shown promising results. Therefore, the present study assessed the levels of this tumor marker in patients with desquamative gingivitis, comparing the CEA level changes before and after treatment with topical corticosteroids.

## Methods

###  Study population and sample size

 This study was prepared based on the STRORB statement.^19^The present case‒control study included the patients presenting to the Oral Medicine Department of Tabriz Faculty of Dentistry with desquamative gingivitis in OLR background as the case group in 2020. The control group was selected from healthy individuals without any oral diseases. The control group was matched with the case group for age and gender.

 The sample size calculation was performed based on a study by Rhodus et al.^20^ Considering an effect size of δ = 5, a standard deviation (SD) of σ = 7.5, a statistical power of 80%, and an error rate of 5%, 20 samples were selected for each group.

 The exclusion criteria included diseases and conditions that could affect CEA levels, including gastrointestinal malignancies, breast, pancreas, gastric, and hepatic cancers, other precancerous lesions, smoking, immune system disorders such as AIDS, chemotherapy, being under treatment for desquamative gingivitis within the past two months, and pregnancy.

###  Saliva sampling and assessment of CEA

 Saliva sampling was performed from 9 to 11 am. The participants were asked to avoid eating and drinking for 2 hours before sampling. Two mL of saliva was obtained from each participant and kept in a 15-mL Falcon tube. The CEA assessment was performed using the ELISA method and primary and secondary antibodies of the specific kit (Monobind Inc., Lake Forest, United States). The samples and conjugated solution were added to the ELISA wells for CEA assessment. Then, the wells underwent intubation for 60 minutes; later, they were rinsed, and the pre-prepared substrate was added. After that, the samples were incubated for 15 min. In the last step, the stop solution was added, and the optical density was measured at 450 nm using the reference wavelength of 630 nm. Finally, the results were compared with the standard curve and reported quantitatively in the 5‒250-ng/mL range.

###  Case group treatment

 All the patients in the case group underwent a biopsy procedure, and the samples were sent to a pathologist to confirm the diagnosis.

 The salivary CEA levels of the case group were measured at least 10 days before the treatment initiation. Then, the patients were treated with topical corticosteroids as a mouthwash, consisting of 15 betamethasone ampules (4 mg/mL each) in one bottle of aluminum /magnesium (Al-MG) suspension (240 mL). The patients were treated for three weeks and underwent corticosteroid tapering for another three weeks. After the treatment course, the patients underwent another salivary CEA assessment, and the results were compared with the pre-intervention results.

###  Statistical analysis

 The results were reported as frequencies, percentages, and means ± SD. Moreover, the Kolmogorov-Smirnov test was used to assess the data normality. The Mann–Whitney U test was used to compare the CEA levels of case and control groups, and the Kruskal–Wallis test was used to compare the CEA levels of the case group before and after treatment. Data analysis was performed using SPSS 17, and the significance level was considered 0.05.

## Results

 The present study involved 20 patients diagnosed with desquamative gingivitis associated with OLP alongside 20 healthy individuals as a control group. The mean age of the participants in the case group was 50.9 ± 12.8, with 48.9 ± 9.8 in the control group. The case group comprised 9 males and 11 females, and the control group included 11 males and 9 females. The mean ± SD of pre-intervention salivary CEA levels was significantly higher in the case group (174.06 ± 95.55 ng/mL) compared to the control group (55.66 ± 41.62 ng/mL) (*P* < 0.001). Table 1 presents a comparison of initial CEA levels in the case and control groups.

 Furthermore, a comparison of CEA levels before and after treatment in the case group revealed a noteworthy reduction in post-intervention salivary CEA levels. The pre-intervention salivary CEA level (174.06 ± 95.55 ng/mL) was significantly higher than the post-intervention salivary CEA level in the case group (96.77 ± 66.25 ng/mL) (P < 0.001). Table 2 presents the results of the comparison of CEA levels in the saliva of the case group before and after treatment. All data are accessible in Supplementary file 1.

**Table 1:** Comparison of initial CEA (ng/mL) levels in case and control groups

	**Mean±SD**	**Min**	**Max**	***P*** ** value**
Case (n = 20)	174.06 ± 95.55	61.70	296.00	< 0.001
Control (n = 20)	55.66 ± 41.26	4.60	136.80	

CEA: carcinoembryonic antigen, SD: standard deviation The *P* value is based on the independent t-test.

**Table 2:** Comparison of CEA levels in saliva of the case group before and after treatment

**CEA (ng/mL)**	**Mean±SD**	***P*** ** value**
Before treatment	174.06 ± 95.55	< 0.001
After treatment	96.77 ± 66.25	

CEA: carcinoembryonic antigen, SD: standard deviation. The *P* value is based on a paired-sample *t* test.

## Discussion

 Tumor markers are categorized into different types. Some markers are specific to a particular type of cancer, while others can be present in multiple types. Elevated levels of tumor markers alone cannot confirm a cancer diagnosis; however, when combined with specific procedures, tumor marker assessments can serve as valuable diagnostic tools. Tumor markers can aid in the early diagnosis and screening of cancer, disease progression evaluation, treatment effectiveness, and recurrence detection. One such tumor marker is CEA, a multifunctional glycoprotein part of the immunoglobulin superfamily. CEA plays dual regulatory roles in both cancer and fetal development. Due to its significant biological functions in cancer regulation, CEA levels can become abnormally elevated in various cancers.^21^ Consequently, the present study aimed to investigate the salivary levels of CEA in patients with desquamative gingivitis before and after treatment with topical corticosteroids.

 The results of the current study indicated that the pre-intervention salivary levels of CEA were significantly higher in the case group compared to the control group. Zheng et al^17^ reported elevated serum and salivary levels of CEA in oral precancerous and cancerous lesions, including OLP, leukoplakia, and oral SCC. They showed that salivary CEA levels were associated with the clinical stage of the SCC and lymph node metastasis, while serum CEA levels were only associated with lymph node metastasis, suggesting that saliva may serve as a more effective and sensitive diagnostic tool for these conditions.

 Honarmand et al^22^ showed that salivary CEA levels were higher in patients with oral SCC than in the control group. Therefore, salivary levels of CEA can be helpful for the early diagnosis of oral SCC, which is compatible with our results. These findings suggest that the serum CEA may be a potential biomarker for the malignant transformation of OLP.

 The CEA levels decreased after topical corticosteroid therapy in the present study. Similar to this result, some studies have shown the relationship between elevated CEA levels with metastasis and prognosis in patients with multiple tumors. Aggarwal et al^23^ reported that CEA could be a valuable serum marker for monitoring and diagnosing the effectiveness of metastatic colorectal cancer treatment. However, a different conclusion was reached by Grimm et al,^24^ who reported that CEA levels did not change during the course of cancer treatment. The discrepancies observed in the results of these studies could be attributed to the diverse types of cancers that were analyzed. Each type of cancer has unique biological characteristics, progression patterns, and responses to treatment, which may influence the findings. Furthermore, variations in study methodologies and demographic factors could also contribute to the differences in the outcomes.

 Corticosteroids are frequently classified as a symptomatic approach for treating OLRs. However, the notable reduction in CEA levels following corticosteroid therapy in this study suggests that these medications may offer benefits beyond merely alleviating the symptoms. A study by Bindakhil et al^25^ highlighted that the application of topical corticosteroids in the management of OLP not only addresses the immediate discomfort associated with the condition but also has the potential to postpone the onset of cancerous developments.

 This study’s limitation was that the degree of dysplasia in lichenoid reactions was not considered due to a limited number of patients. It is suggested that in future studies, OLR with varying degrees of dysplasia be compared to assess the relationship between salivary CEA and malignant conversion in OLR.

## Conclusion

 Increased levels of ACE in the case group indicated the precancerous nature of desquamative gingivitis associated with OLR. Furthermore, it appears that after topical corticosteroid therapy, CEA levels significantly decreased in patients with desquamative gingivitis.

## Competing Interests

 The authors declare that they have no competing interests.

## Consent for Publication

 Not applicable.

## Data Availability Statement

 The data are presented as supplementary file 1.

## Ethical Approval

 All procedures were in accordance with the ethical standards of the national committee on human experimentation and with the Helsinki Declaration of 1975, as revised in 2013. Study was explained to patients and written detailed informed consent was obtained from all participants. The study was approved by the Ethics Committee of the Tabriz University of Medical Sciences with the ethics code of IR.TBZMED.REC.1399.140.

## Supplementary Files


Supplementary file 1. Data obtained in the research.
